# Healthcare Utilization and Costs in Patients With Somatic Symptom and Related Disorders Compared With Those With Depression and Healthy Controls: A Nationwide Cohort Study

**DOI:** 10.1155/da/8352965

**Published:** 2024-11-28

**Authors:** Jun Ho Seo, Minkyung Han, Sunghyuk Kang, Se Joo Kim, Inkyung Jung, Jee In Kang

**Affiliations:** ^1^Department of Psychiatry, Yonsei University Wonju College of Medicine, Wonju, Republic of Korea; ^2^Institute of Behavioral Science in Medicine, Yonsei University College of Medicine, Seoul, Republic of Korea; ^3^Biostatistics Collaboration Unit, Department of Biomedical Systems Informatics, Yonsei University College of Medicine, Seoul, Republic of Korea; ^4^Department of Preventive Medicine, Yonsei University College of Medicine, Seoul, Republic of Korea; ^5^Department of Psychiatry, Yonsei University College of Medicine, Seoul, Republic of Korea; ^6^Division of Biostatistics, Department of Biomedical Systems Informatics, Yonsei University College of Medicine, Seoul, Republic of Korea

**Keywords:** health seeking behavior, healthcare utilization, medical cost, somatic symptom and related disorder, somatization disorder, somatoform disorders

## Abstract

**Introduction:** Patients with somatic symptom and related disorders (SSRDs) often face diagnostic delays, leading to frustration, unnecessary medical procedures, and excessive costs. This study examines healthcare utilization and costs in the 3 years before diagnosing SSRDs, comparing them to patients with depressive disorders and individuals with no mental disorder using data from the Korean National Health Insurance claims database. The analysis also addresses the influence of medical comorbidities by focusing on patients without them.

**Methods:** Utilizing Korean nationwide medical claims database covering all South Koreans, we identified individuals aged 15–64 diagnosed with SSRDs between 2015 and 2019. A corresponding group diagnosed with depression served as controls for nonpsychotic mental disorders. We analyzed medical costs and healthcare utilization comparing the SSRDs group to the depression group and the group with no mental disorder using nonparametric tests, including a specific analysis for those with a Charlson Comorbidity Index (CCI) of zero.

**Results:** The study encompassed 84,223 SSRD patients, 336,919 with depressive disorders, and 269,444 individuals with no mental disorder. Patients with SSRDs had significantly higher healthcare costs and made more frequent use of outpatient and emergency services than both control groups, a pattern consistent even in patients without medical comorbidities.

**Conclusion:** This large nationwide cohort study confirmed that patients with SSRDs frequently used the healthcare system and incurred considerable costs before their diagnosis. The findings suggest that plans for early recognition and intervention, along with mental health support for this population, are urgently needed to assist them and improve the efficiency of the healthcare system.

## 1. Introduction

Somatic symptom and related disorders (SSRDs) are characterized by distressing somatic symptoms and concerns which are difficult to explain by diagnosable medical conditions. The symptoms often have no identifiable medical basis and are inconsistent with medical examinations [1]. While most medically unexplained physical symptoms eventually resolve, some develop into chronic, disabling somatic conditions [2, 3]. These somatic symptoms are considered to arise from or be influenced by emotional distress, even though many SSRD patients attribute their symptoms solely to undiscovered medical causes [4]. Consequently, patients with SSRDs often experience worry and frustration due to delays in receiving appropriate diagnoses and psychiatric engagement [5], and they are repeatedly exposed to unnecessary medical examinations or treatments [6]. This frequent use of the healthcare system, often accompanied by dissatisfaction and self-perceptions of serious illness, has been observed clinically [7–9]. Previous studies have reported that patients with somatization utilize more healthcare resources, including both outpatient and inpatient services, and incur higher medical costs than patients without somatization [10–15]. Despite the relatively high prevalence rates of SSRDs [16], early detection and engagement of these patients in appropriate psychiatric care remain challenging. Moreover, there is a scarcity of real-world evidence at a national level to support the need for interventions in this population.

The Korean Health Insurance Review and Assessment Service (HIRA) database offers an opportunity to study healthcare usage and costs using large-scale real-world data. The Korean National Health Insurance Service (NHIS) is a compulsory social insurance system to which all medical care providers in the Republic of Korea are contracted, thereby covering the entire population [15]. The HIRA database includes data on all NHIS claims, featuring demographic characteristics, diagnostic codes by the International Classification of Diseases, 10th Revision (ICD-10), types of healthcare, costs, and prescribed medications and procedures for every healthcare utilization [17]. Using the HIRA database enables to identify all patients with a specific diagnosis and confirm all medical comorbidities of each patient with date information. Furthermore, as all Korean citizens are assigned by a 13-digit resident registration number from birth to death—which is also used by all Korean hospitals and clinics to register individual patients in the medical insurance system—the risk of overlapping medical records is minimal, even if a patient relocates.

By using Korean nationwide cohort data from the HIRA database, the present study aims to investigate the patterns of healthcare use and costs of SSRD patients. Firstly, we aim to explore the sociodemographic characteristics of SSRD patients. Secondly, we aim to examine the medical use patterns of SSRD patients during the 3 years before the initial diagnosis of SSRD, particularly in terms of nonpsychiatric healthcare use and costs, compared to those of a depressive disorder group and a group with no mental disorder. Lastly, we aim to confirm distinctive features of the SSRD group by controlling for possible confounders such as age, sex, and medical comorbidities.

## 2. Methods

### 2.1. Data Source

This study utilized the HIRA database of Korean nationwide medical claims from 2012 to 2019. The current study was approved by HIRA (M20220112767) and the Institutional Review Board of Severance Hospital, Seoul, Republic of Korea (4-2022-0186). The study followed the principles of the Declaration of Helsinki [18]. Data are anonymized.

### 2.2. Selection of Case and Control Groups

For the case group, patients with a first principal diagnosis of SSRDs according to ICD-10 codes F45.x (excluding F45.22, F45.3, F45.8) from January 1, 2012, to December 31, 2019 were identified. Exclusions were made to secure diagnostic validity of the case group: F45.3 (somatoform autonomic dysfunction) and F45.8 (other somatoform disorders) were excluded because the two codes were frequently diagnosed by nonpsychiatric clinics. F45.22 (body dysmorphic disorder) was excluded because it is not classified under SSRDs [19]. A washout period of 3 years ensured that patients included had no principal or additional diagnosis of SSRDs at least 3 years prior to the index date. Thus, patients newly diagnosed with SSRDs between 2015 and 2019 were designated as the case group.

For the mental disorder control group, depressive disorders were selected as the primary nonpsychotic mental disorder for comparison, given their known association with significant healthcare utilization. This group was defined as patients with depressive disorders (excluding any SSRDs) diagnosed using codes F32.1 (moderate depressive episode), F32.2 (severe depressive episode without psychotic symptoms), or F33.x (recurrent depressive disorders) from January 1, 2012, to December 31, 2019. Codes F32.0 (mild depressive episode), F32.8 (other depressive episodes), and F32.9 (unspecified depressive episode) were excluded to maintain diagnostic validity. Similar to the case group, a 3-year washout period was applied, identifying patients with newly diagnosed depressive disorders between 2015 and 2019. Next, among patients with newly diagnosed depressive disorders between 2015 and 2019, patients who received a diagnosis of any SSRDs between 2012 and 2019 were excluded. In this way, patients with newly diagnosed depressive disorders between 2015 and 2019 without any SSRDs were set as the depression group. The control group with no mental disorder comprised subjects coded with a Z00.8 (other general examinations) or Z00.0 (general medical examination) from January 1, 2015, to December 31, 2019, without any F codes (any mental disorders) during 2012–2019. Both case and control groups included subjects aged 15–64 years.

### 2.3. Demographic and Clinical Characteristics

Demographic data collected for all subjects included sex, age at diagnosis, and Charlson Comorbidity Index (CCI) scores. Subjects were categorized by CCI score as 0, 1, 2, or ≥3 based on ICD-10 codes [20]. Healthcare utilization outcomes examined included the number of hospitalizations, outpatient department (OPD) visits, and emergency department (ED) visits for both psychiatric and nonpsychiatric reasons during the 3 years before the index diagnosis. Additionally, medical costs per patient for both psychiatric and nonpsychiatric healthcare uses during this period were assessed. Visits with a primary diagnosis of any mental disorder were considered psychiatric healthcare visits.

### 2.4. Statistical Analysis

Healthcare utilization costs and the number of visits between the case and depression group, and between the case and the group with no mental disorder, were compared using Wilcoxon ranked sum test, given the nonnormal distribution of all variables. To control for potential confounding effects, a 1:1 : 1 case–control matching based on sex and exact age was performed for 83,874 newly diagnosed SSRD patients, 83,874 depressive disorder patients, and 83,874 individuals with no mental disorder. Healthcare system utilization variables for these matched groups were compared using Wilcoxon signed-rank test. A sensitivity analysis for a subset with a CCI score of 0 was conducted in the same manner. All tests were two-sided, with a significance threshold set at *p*  < 0.05. Statistical analyses were conducted from July 4, 2022, to September 30, 2022, using SAS Enterprise Guide, version 7.1 (SAS Institute). All medical costs were presented as United States Dollars (USD).

## 3. Results

### 3.1. Demographics and Baseline Characteristics

**Figure 1:**
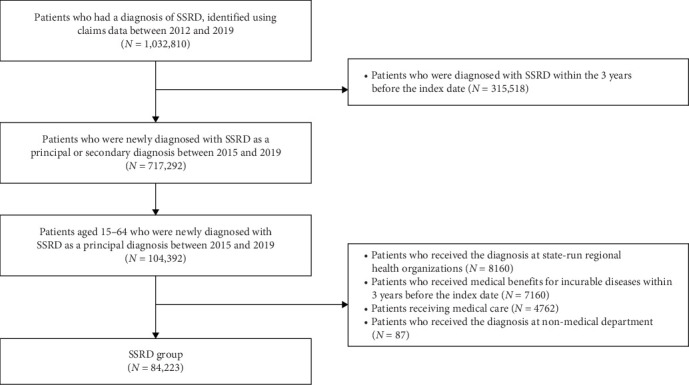
Flow of case population selection. SSRD, somatic symptom and related disorder.

**Figure 2:**
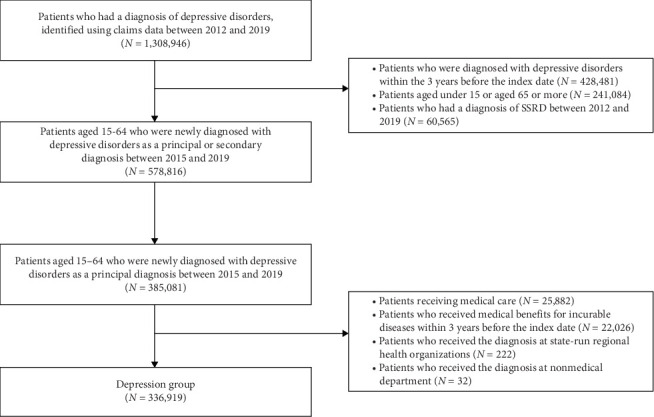
Flow of mental disorder control population selection.

From the HIRA database, we identified 104,392 patients aged 15–64 newly diagnosed with SSRDs and 385,081 patients aged 15–64 with newly diagnosed depressive disorders (excluding SSRDs) between January 2015 and December 2019, after applying a washout period from January 2012 to December 2014. Patients receiving medical care or medical benefits for rare incurable diseases were excluded from the analysis due to their potential to influence the frequency of medical system use. Detailed flowcharts for the SSRD and depressive disorder groups are presented in Figures 1 and 2.

**Figure 3:**
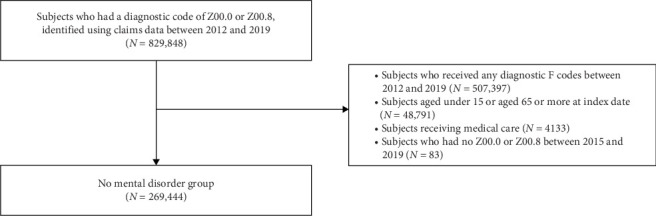
Flow of no mental disorder population selection.

After all, the analysis included 84,223 patients with SSRDs (median [interquartile range—IQR] age, 48 [36–57] years; 52,459 females [62.3%] and 31,764 males [37.7%]), and 336,919 patients with depression (median [IQR] age, 38 [27–51] years; 211,829 females [62.9%] and 125,090 males [37.1%]). The group with no mental disorder included 269,444 subjects (median [IQR] age, 44 [33–53] years; 150,349 females [55.8%] and 119,095 males [44.2%]), after excluding 4133 recipients of medical care or benefits. The selection flowchart for the group with no mental disorder is shown in Figure 3. In terms of the CCI score, patients with SSRDs had significantly higher scores than those in the depression or individuals with no mental disorder, while patients with depression and individuals with no mental disorder had a similar range of scores (median [IQR [3, 21] for SSRDs, 1 [0–2] for both depression and individuals with no mental disorder). Detailed demographic characteristics are presented in Table 1.

### 3.2. Healthcare Utilization Before and After Age-Sex Matching

Regarding healthcare utilization costs, patients with SSRDs had a significantly higher median [IQR] total medical cost per patient of $1645.1 [725.8–3360.5] (mean [SD], $2,720.6 [4,924.8]) during the 3 years before diagnosis compared to both individuals with no mental disorder (median [IQR], $742.6 [315.7–1731.4]; mean [SD], $1629.7 [4607.2]) and patients with depression (median [IQR], $1264.0 [527.4–2781.7]; mean [SD], $2350.2 [4,941.4]) (both *p*  < 0.001). Excluding psychiatric healthcare utilization, the SSRDs group still showed significantly higher median [IQR] nonpsychiatric medical cost per patient of $1502.2 [665.9–3082.8] compared to depression group ($1039.7 [429.1–2384.3]) (*p*  < 0.001). For the number of nonpsychiatric healthcare uses, the SSRDs group had higher OPD and ED visits than both the depression group and the group with no mental disorder. Moreover, patients with SSRDs were more frequently hospitalized for nonpsychiatric causes than both comparison groups. Detailed information is provided in Table 2.

After matching for age and sex between groups (*N* = 83,874 for all groups), the trends remained consistent. Patients with SSRDs still showed a higher median [IQR] total medical costs per patient of $1647.4 [726.4–3363.7] (mean [SD], $2723.6 [4932.8]) compared to both individuals with no mental disorder (median [IQR], $795.1 [339.4–1790.3]; mean [SD], $1699.2 [4447.6]) and patients with depression (median [IQR], $1509.7 [638.9–3186.1]; mean [SD], $2711.9 [5516.9]) (both *p*  < 0.001). In terms of nonpsychiatric medical costs, patients with SSRDs incurred higher expenses than those with depression (median [IQR], $1504.9 [666.4–3086.6] vs. $1261.9 [526.8–2780.3], respectively) (*p*  < 0.001). In addition, the SSRDs group showed higher numbers of nonpsychiatric healthcare uses in all settings including OPD, ED, and hospitalizations compared to both the depression group and the group with no mental disorder. Detailed information is available in Table 3.

Additionally, subgroup analyses for older or younger subjects (divided by median age) and by sex showed similar results to the primary analysis, indicating that the SSRDs group incurred significantly higher medical costs per patient than the depression group or the group with no mental disorder. Results for subgroup analyses by age or sex are available in the supporting information (Tables S1, S2, S3, S4).

### 3.3. Healthcare Utilization in a Subset of Subjects With CCI Score 0

In an age-sex matched subset of subjects with a CCI score of 0 (*N* = 16,241 for all groups), patients with SSRDs demonstrated a higher median [IQR] total medical cost per patient of $575.0 [244.5–1324.4] compared to individuals with no mental disorder (median [IQR], $365.9 [149.0–849.4], *p*  < 0.001). However, there was no significant difference in the median total medical cost per patient between the SSRDs group and the depression group (median [IQR], $546.7 [210.7–1314.4] for the depression group) (*p* = 0.124). Meanwhile, for nonpsychiatric medical costs, patients with SSRDs incurred higher costs ($513.1 [220.7–1135.0]) compared to both the depression group ($421.4 [157.3–999.2]) and the group with no mental disorder ($365.9 [149.0–849.4]) (both *p*  < 0.001). For number of nonpsychiatric healthcare uses, patients with SSRDs had higher numbers of OPD visits than those in both the depression group and the group with no mental disorder; however, the numbers of ED visits and hospitalizations were not significantly different between the SSRDs group and the depression group. Detailed information can be found in Table 4.

## 4. Discussion

The present study found that patients with SSRDs used the healthcare system more frequently and incurred substantially higher costs during the 3 years preceding their initial diagnosis compared to both the depression group and the group with no mental disorder, in a large-scale nationwide cohort. This pattern persisted even in a subset of the population without medical comorbidities, suggesting that high healthcare utilization and associated costs in SSRD patients are largely independent of depression or other medical comorbidities.

Patients with SSRDs had a greater number of mental-health-not-related visits across all settings—outpatient clinics, inpatient care, and EDs—resulting in much higher medical costs per patient compared to individuals with no mental disorder. This finding aligns with previous studies that investigated medical utilization and costs in different settings and with various methodologies. For instance, a study conducted in primary healthcare settings reported that patients provisionally diagnosed with somatoform disorders through self-report questionnaires had more frequent visits to primary care and specialists, as well as more ED visits, hospitalizations, and higher costs in both inpatient and outpatient settings compared to patients without somatoform disorders [21]. Another study found that patients identified through multiple screening instruments as having somatization had twice the healthcare utilization of nonsomatizing patients, with costs amounting to about $1500 per year in the outpatient setting [22]. In addition, a study targeting entire SSRDs by using structured interviews also showed that patients with somatoform disorder had more than twice the primary care visits of controls [23]. While many previous studies defined case subjects by provisional diagnosis using self-report questionnaires such as somatic symptom inventory or Patient Health Questionnaire somatoform disorder module (PHQ-15) [10–15, 21, 22], a handful of large-scale studies using clinical diagnoses in real-world settings have shown similar trends. For example, a large-scale retrospective cohort study in Germany using clinical diagnosis for patients with functional somatic syndromes, including somatoform disorders, found that these patients experienced costly diagnostic tests and surgeries more frequently than patients without these syndromes [24]. Given the variability in reported prevalence rates of SSRDs depending on the methodology used for diagnosis [25], it is crucial to study the characteristics of healthcare usage charecteristics among patients with SSRDs diagnosed both provisonally and clinically. Thus, the similar results across studies—whether defining SSRDs through clinical diagnosis in real-world settings, including this study, or through provisional diagnosis for somatoform disorders—suggest that somatization may be a key factor in increased healthcare use and costs.

Notably, this study also found that SSRD patients had higher nonpsychiatric medical costs and more frequent healthcare visits compared to the depression group. The result supports that SSRD patients' extensive healthcare utilization stems from the unique characteristics of SSRDs, beyond those of depression. Previously, there have been concerns that depression or other anxiety features highly comorbid with SSRD patients rather than features of SSRD itself might influence healthcare usage in SSRD patients [22]. As multiple literatures have reported increased healthcare utilization in patients with depressive or anxiety disorders [26–29], most studies for SSRDs lacking a mental disorder control group have struggled to conclusively attribute the frequent healthcare usage of SSRD patients to their specific somatization-related features. In response to this concern, some studies have investigated medical cost and healthcare usage of somatizing patients with controlling comorbid depression or anxiety. A 5-year follow-up study in general population reported that somatization and depression were independently related with medical costs in both inpatient and outpatient settings [30]. In the study, baseline somatization predicted elevated medical costs more than baseline depression predicted. Similarly, a Danish register-based study reported that patients with somatoform disorders had higher healthcare usage than patients with anxiety disorders and controls in both inpatient and outpatient settings, despite the small sample size [23]. Consistently, the current study confirmed similar results at a national level evidence by using mental disorder control group with depressive disorders.

Meanwhile, the current results do not necessarily imply that depression has no role in the healthcare utilization of patients with SSRDs. Patients with depression also showed greater use of healthcare services compared to individuals with no mental disorder. In addition, in this study, the differences in healthcare usage between patients with SSRDs and the depression group were more prominent in outpatient settings. A previous study also showed a similar pattern, indicating that somatization was strongly associated with outpatient visits and costs, whereas depression had a major impact on inpatient visits and costs [23]. It seems that individual's health-seeking behavior and healthcare use are affected by both somatization and depression, albeit in slightly different aspects and to varying degrees.

Moreover, the findings in subjects with a zero CCI score, which showed SSRD patients incurring higher medical costs and using healthcare services more frequently than those with depressive disorders, imply that the health-seeking behaviors leading to these high costs and frequent healthcare uses are independent of medical comorbidities. It is expected that this subgroup with a CCI score of 0 among the SSRD population exhibits more intrinsic features of SSRDs, considering that they were diagnosed without specific underlying medical conditions. Therefore, SSRD patients with a CCI score of 0 can be instrumental in developing screening processes to identify characteristics of potential SSRD patients. Future studies should aim to develop a screening process that can distinguish potential SSRD patients among those with medically unexplained physical symptoms in nonpsychiatric healthcare settings. By recognizing potential SSRD patients earlier, we can provide timely mental health support and prevent the costly overuse of nonmental health services, thereby reducing their exposure to increased morbidity due to frequent misdiagnoses and excessive investigations [31], improving their quality of life and health outcomes, and enhancing the efficiency of the healthcare system.

The current study has a strength in the reliability of its results, derived from using a very large sample size with nationwide cohort data that encompasses the entire population. In addition, the conservative definition of depression as the primary nonpsychotic mental disorder comparison group enables clearer distinctions in the findings specific to SSRDs. Furthermore, similar results from a sensitivity analysis with a subset of subjects with a zero CCI score reinforce the conclusion. However, there are some limitations to this study. First, subjects receiving medical care or medical benefits for rare incurable diseases were excluded from the analyses. Although this exclusion was necessary to control confounding effects, the results cannot consequently be generalized to people with very low socioeconomic status. Second, we did not investigate if there are any differences according to specific diagnoses within SSRDs. As SSRDs encompass heterogenous diagnostic groups, the current results cannot be uniformly applied to all diagnoses within SSRDs. Third, the year of the index date for each diagnosis was not adjusted. However, this can be compensated for, as the study investigated healthcare uses and costs over a considerable period of 3 years before the index diagnosis.

## 5. Conclusion

This large nationwide cohort study confirmed that SSRD patients frequently utilize the healthcare system and incur considerable costs before diagnosis. The findings underscore the need for early recognition and intervention, coupled with mental health support, to assist these patients and enhance the efficiency of the healthcare system.

## Figures and Tables

**Table 1:** Baseline characteristics of subjects in each group of somatic symptom and related disorders, depressive disorders, and the group with no mental disorder.

SSRDs group (*N* = 84,223)		Depression group (*N* = 336,919)	No mental disorder group (*N* = 269,444)
Sex, *N* (%)			
Male	31,764 (37.7)	125,090 (37.1)	119,095 (44.2)
Female	52,459 (62.3)	211,829 (62.9)	150,349 (55.8)
Age, *N* (%)			
Median (IQR)	48 (36–57)	38 (27–51)	44 (33–53)
Mean ± SD	45.7 ± 13.1	39.1 ± 13.7	42.8 ± 12.7
≥15, <20	2296 (2.7)	18,731 (5.6)	10,993 (4.1)
≥20, <25	5229 (6.2)	48,035 (14.3)	18,038 (6.7)
≥25, <30	5675 (6.7)	38,233 (11.4)	19,727 (7.3)
≥30, <35	5965 (7.1)	35,326 (10.5)	26,597 (9.9)
≥35, <40	7142 (8.5)	37,108 (11.0)	27,773 (10.3)
≥40, <45	8277 (9.8)	32,822 (9.7)	37,881 (14.1)
≥45, <50	10,203 (12.1)	32,849 (9.7)	34,739 (12.9)
≥50, <55	11,968 (14.2)	31,984 (9.5)	35,060 (13.0)
≥55, <60	14,332 (17.0)	33,719 (10.0)	31,733 (11.8)
≥60, <65	13,136 (15.6)	28,112 (8.3)	26,903 (10.0)
Index date year, *N* (%)			
2015	17,588 (20.9)	58,844 (17.5)	36,930 (13.7)
2016	17,994 (21.4)	63,363 (18.8)	45,070 (16.7)
2017	16,659 (19.8)	65,375 (19.4)	48,085 (17.9)
2018	16,279 (19.3)	72,701 (21.6)	62,510 (23.2)
2019	15,703 (18.6)	76,636 (22.7)	76,849 (28.5)
CCI score, *N* (%)			
Median (IQR)	2 (1–3)	1 (0–2)	1 (0–2)
Mean ± SD	2.0 ± 1.7	1.5 ± 1.5	1.2 ± 1.4
0	16,260 (19.3)	96,598 (28.7)	98,973 (36.7)
1	21,535 (25.6)	99,837 (29.6)	84,779 (31.5)
2	18,767 (22.3)	68,497 (20.3)	47,152 (17.5)
≥3	27,661 (32.8)	71,987 (21.4)	38,540 (14.3)

Abbreviations: IQR, interquartile range; SD, standard deviation; SSRDs, somatic symptom and related disorders.

**Table 2:** Healthcare system utilization costs and visit numbers in the 3 years before initial diagnosis: comparison among patients with somatic symptom and related disorders, depressive disorders, and individuals with no mental disorder.

Healthcare system utilization	SSRDs group	Depression group		No mental disorder group	
	(*N* = 84,223)	(*N* = 336,919)	*p* Value	(*N* = 269,444)	*p* Value
Medical cost ($) per patient, median (IQR)					
Costs by all uses	1645.1 (725.8–3360.5)	1264.0 (527.4–2781.7)	<0.001	742.6 (315.7–1731.4)	<0.001
Mean ± SD	2720.6 ± 4924.8	2350.2 ± 4941.4	—	1629.7 ± 4607.2	—
Costs by nonpsychiatric uses	1502.2 (665.9–3082.8)	1039.7 (429.1–2384.3)	<0.001	742.6 (315.7–1731.4)	<0.001
Mean ± SD	2488.9 ± 4637.6	2002.6 ± 4460.8	—	1629.7 ± 4607.2	—
Visit number of outpatient clinic use, median (IQR)					
All-cause visits	47 (24–82)	34 (17–61)	<0.001	24 (12–42)	<0.001
Mean ± SD	62.76 ± 62.07	46.81 ± 47.78	—	30.93 ± 28.90	—
Mental-health-not-related visits	44 (23–76)	30 (15–55)	<0.001	24 (12–42)	<0.001
Mean ± SD	58.94 ± 59.00	42.11 ± 44.75	—	30.93 ± 28.91	—
Visit number of acute care use, median (IQR)					
All-cause visits					
Emergency department	0 (0–0)	0 (0–0)	<0.001	0 (0–0)	<0.001
Mean ± SD	0.09 ± 0.48	0.08 ± 0.37	—	0.03 ± 0.18	—
0	78,507 (93.2%)	316,027 (93.8%)	—	263,029 (97.6%)	—
≥1	5,716 (6.8%)	20,892 (6.2%)	—	6,415 (2.4%)	—
Inpatient	0 (0–1)	0 (0–1)	<0.001	0 (0–1)	<0.001
Mean ± SD	0.76 ± 1.48	0.68 ± 1.43	—	0.39 ± 0.89	—
0	50,886 (60.4%)	212,067 (62.9%)	—	198,125 (73.5%)	—
≥1	33,337 (39.6%)	124,852 (37.1%)	—	71,319 (26.5%)	—
Mental-health-not-related visits					
Emergency department	0 (0–0)	0 (0–0)	<0.001	0 (0–0)	<0.001
Mean ± SD	0.09 ± 0.47	0.07 ± 0.36	—	0.03 ± 0.18	—
0	78,683 (93.4%)	317,294 (94.2%)	—	263,029 (97.6%)	—
≥1	5,540 (6.6%)	19,625 (5.8%)	—	6,415 (2.4%)	—
Inpatient	0 (0–1)	0 (0–1)	<0.001	0 (0–1)	<0.001
Mean ± SD	0.75 ± 1.47	0.66 ± 1.42	—	0.39 ± 0.89	—
0	51,360 (61.0%)	215,739 (64.0%)	—	198,125 (73.5%)	—
≥1	32,863 (39.0%)	121,180 (36.0%)	—	71,319 (26.5%)	—

Abbreviations: IQR, interquartile range; SD, standard deviation; SSRDs, somatic symptom and related disorders.

**Table 3:** Healthcare system utilization costs and visit numbers in the 3 years before initial diagnosis: comparison among patients with somatic symptom and related disorders, depressive disorders, and individuals with no mental disorder, among age-sex matched samples.

Healthcare system utilization	SSRDs group	Depression group		No mental disorder group	
	(*N* = 83,874)	(*N* = 83,874)	*p* Value	(*N* = 83,874)	*p* Value
Medical cost ($) per patient, median (IQR)					
Costs by all uses	1647.4 (726.4–3363.7)	1509.7 (638.9–3186.1)	<0.001	795.1 (339.4–1790.3)	<0.001
Mean ± SD	2723.6 ± 4932.8	2711.9 ± 5516.9	—	1699.2 ± 4447.6	—
Costs by nonpsychiatric uses	1504.9 (666.4–3086.6)	1261.9 (526.8–2780.3)	<0.001	795.1 (339.4–1790.3)	<0.001
Mean ± SD	2492.3 ± 4645.5	2341.6 ± 5028.2	—	1699.2 ± 4447.6	—
Visit number of outpatient clinic use, median (IQR)					
All-cause visits	47 (24–82)	40 (20–72)	<0.001	26 (13–45)	<0.001
Mean ± SD	62.80 ± 62.12	54.55 ± 54.89	—	33.20 ± 30.93	—
Mental-health-not-related visits	44 (23–77)	36 (17–65)	<0.001	26 (13–45)	<0.001
Mean ± SD	58.99 ± 59.07	49.48 ± 51.75	—	33.20 ± 30.93	—
Visit number of acute care use, median (IQR)					
All-cause visits					
Emergency department	0 (0–0)	0 (0–0)	<0.001	0 (0–0)	<0.001
Mean ± SD	0.09 ± 0.48	0.07 ± 0.34	—	0.03 ± 0.18	—
0	78,179 (93.2%)	78,773 (93.9%)	—	81,893 (97.6%)	—
≥1	5,695 (6.8%)	5,101 (6.1%)	—	1981 (2.4%)	—
Inpatient	0 (0–1)	0 (0–1)	0.057	0 (0–1)	<0.001
Mean ± SD	0.76 ± 1.48	0.75 ± 1.54	—	0.39 ± 0.93	—
0	50,667 (60.4%)	50,868 (60.7%)	—	62,153 (74.1%)	—
≥1	33,207 (39.6%)	33,006 (39.3%)	—	21,721 (25.9%)	—
Mental-health-not-related visits					
Emergency department	0 (0–0)	0 (0–0)	<0.001	0 (0–0)	<0.001
Mean ± SD	0.09 ± 0.47	0.07 ± 0.32	—	0.03 ± 0.18	—
0	78,355 (93.4%)	79,046 (94.2%)	—	81,893 (97.6%)	—
≥1	5,519 (6.6%)	4,828 (5.8%)	—	1981 (2.4%)	—
Inpatient	0 (0–1)	0 (0–1)	<0.001	0 (0–1)	<0.001
Mean ± SD	0.75 ± 1.47	0.73 ± 1.52	—	0.39 ± 0.93	—
0	51,139 (65.6%)	51,765 (61.7%)	—	62,153 (74.1%)	—
≥1	32,735 (34.4%)	32,109 (38.3%)	—	21,721 (25.9%)	—

Abbreviations: IQR, interquartile range; SD, standard deviation; SSRDs, somatic symptom and related disorders.

**Table 4:** Healthcare system utilization costs and visit numbers in the 3 years before initial diagnosis: comparison among patients with somatic symptom and related disorders, depressive disorders, and individuals with no mental disorder in subjects with CCI score 0 of age-sex matched samples.

Healthcare system utilization	SSRDs group	Depression group		No mental disorder group	
	(*N* = 16,241)	(*N* = 16,241)	*p* Value	(*N* = 16,241)	*p* Value
Medical cost ($) per patient, median (IQR)					
Costs by all uses	575.0 (244.5–1324.4)	546.7 (210.7–1314.4)	0.124	365.9 (149.0–849.4)	<0.001
Mean ± SD	1108.3 ± 3160.4	1106.2 ± 1995.1	—	777.6 ± 1379.8	—
Costs by nonpsychiatric uses	513.1 (220.7–1135.0)	421.4 (157.3–999.2)	<0.001	365.9 (149.0–849.4)	<0.001
Mean ± SD	973.0 ± 3008.4	862.1 ± 1358.4	—	777.6 ± 1379.8	—
Visit number of outpatient clinic use, median (IQR)					
All-cause visits	19 (9–37)	17 (7–33)	<0.001	14 (6–26)	<0.001
Mean ± SD	27.74 ± 28.80	24.58 ± 25.82	—	18.77 ± 19.39	—
Mental-health-not-related visits	18 (9–34)	14 (6–28)	<0.001	14 (6–26)	<0.001
Mean ± SD	25.47 ± 26.74	20.85 ± 22.84	—	18.77 ± 19.39	—
Visit number of acute care use, median (IQR)					
All-cause visits					
Emergency department	0 (0–0)	0 (0–0)	0.095	0 (0–0)	<0.001
Mean ± SD	0.03 ± 0.21	0.04 ± 0.22	—	0.01 ± 0.13	—
0	15,741 (96.9%)	15,686 (96.6%)	—	16,037 (98.7%)	—
≥1	500 (3.1%)	555 (3.4%)	—	204 (1.3%)	—
Inpatient	0 (0–0)	0 (0–0)	0.262	0 (0–0)	<0.001
Mean ± SD	0.30 ± 0.74	0.30 ± 0.71	—	0.21 ± 0.55	—
0	12,783 (78.7%)	12,704 (78.2%)	—	13,500 (83.1%)	—
≥1	3458 (21.3%)	3537 (21.8%)	—	2741 (16.9%)	—
Mental-health-not-related visits					
Emergency department	0 (0–0)	0 (0–0)	0.283	0 (0–0)	<0.001
Mean ± SD	0.03 ± 0.20	0.04 ± 0.21	—	0.01 ± 0.13	—
0	15,761 (97.0%)	15,730 (96.9%)	—	16,037 (98.7%)	—
≥1	480 (3.0%)	511 (3.1%)	—	204 (1.3%)	—
Inpatient	0 (0–0)	0 (0–0)	0.989	0 (0–0)	<0.001
Mean ± SD	0.29 ± 0.73	0.29 ± 0.70	—	0.21 ± 0.55	—
0	12,866 (79.2%)	12,872 (79.3%)	—	13,500 (83.1%)	—
≥1	3375 (20.8%)	3369 (20.7%)	—	2741 (16.9%)	—

Abbreviations: CCI, Charlson Comorbidity Index; IQR, interquartile range; SD, standard deviation; SSRDs, somatic symptom and related disorders.

## Data Availability

The datasets generated and/or analyzed during the current study are not publicly available due to Data Protection Laws and Regulations in Korea, but final analyzing results are available from the corresponding authors upon reasonable request.

## Supporting Information

Supporting Information Table S1. Healthcare System Utilization Costs and Visit Numbers in the 3 Years Before Initial Diagnosis: Comparison Among Patients with Somatic Symptom and Related Disorders, Depressive Disorders, and Individuals with no Mental Disorder in the age-sex matched samples, aged 48 (median age) and younger. Table S2. Healthcare System Utilization Costs and Visit Numbers in the 3 Years Before Initial Diagnosis: Comparison Among Patients with Somatic Symptom and Related Disorders, Depressive Disorders, and Individuals with no Mental Disorder in the age-sex matched samples, aged above 48 (median age). Table S3. Healthcare System Utilization Costs and Visit Numbers in the 3 Years Before Initial Diagnosis: Comparison Among Patients with Somatic Symptom and Related Disorders, Depressive Disorders, and Individuals with no Mental Disorder in the age-sex matched samples, men. Table S4. Healthcare System Utilization Costs and Visit Numbers in the 3 Years Before Initial Diagnosis: Comparison Among Patients with Somatic Symptom and Related Disorders, Depressive Disorders, and Individuals with no Mental Disorder in the age-sex matched samples, Women.
