# Supplementary material for: Healthcare Utilization and Costs in Patients With Somatic Symptom and Related Disorders Compared With Those With Depression and Healthy Controls: A Nationwide Cohort Study
Source: Depress Anxiety. 2024 Nov 28;2024:8352965. doi: 10.1155/da/8352965 (PMC11918987; doi:10.1155/da/8352965)
Supplement: Supporting Information — Table S1. Healthcare System Utilization Costs and Visit Numbers in the 3 Years Before Initial Diagnosis: Comparison Among Patients with Somatic Symptom and Related Disorders, Depressive Disorders, and Individuals with no Mental Disorder in the age-sex matched samples, aged 48 (median age) and younger. Table S2. Healthcare System Utilization Costs and Visit Numbers in the 3 Years Before Initial Diagnosis: Comparison Among Patients with Somatic Symptom and Related Disorders, Depressive Disorders, and Individuals with no Mental Disorder in the age-sex matched samples, aged above 48 (median age). Table S3. Healthcare System Utilization Costs and Visit Numbers in the 3 Years Before Initial Diagnosis: Comparison Among Patients with Somatic Symptom and Related Disorders, Depressive Disorders, and Individuals with no Mental Disorder in the age-sex matched samples, men. Table S4. Healthcare System Utilization Costs and Visit Numbers in the 3 Years Before Initial Diagnosis: Comparison Among Patients with Somatic Symptom and Related Disorders, Depressive Disorders, and Individuals with no Mental Disorder in the age-sex matched samples, Women. [file 8352965.f1.docx]

**Supplementary Information**

**Table S1.** **Healthcare System Utilization Costs and Visit Numbers in the Three Years Before Initial Diagnosis: Comparison Among Patients with Somatic Symptom and Related Disorders, Depressive Disorders, and Individuals with no Mental Disorder in the age-sex matched samples, aged 48 (median age) and younger**

|  | | **SSRDs group** | **Depression group** |  | **No mental disorder group** | |
| --- | --- | --- | --- | --- | --- | --- |
| Healthcare system utilization | | (N = 42,242) | (N = 42,242) | P value | (N = 42,242) | P value |
| **Medical cost** ($) **per patient,** median (IQR) | | | | | | |
| **Costs by all uses** | | 1216.8 (525.2-2650.3) | 1064.8 (455.7-2372.7) | <0.001 | 603.2 (258.4-1441.0) | <0.001 |
|  | mean±SD | 2159.3 ±4269.2 | 2027.8 ±5689.3 |  | 1381.8 ±4463.9 |  |
| **Costs by non-psychiatric uses** | | 1095.6 (481.0-2369.7) | 854.4 (366.8-1977.7) | <0.001 | 603.2 (258.4-1441.0) | <0.001 |
|  | mean±SD | 1927.4 ±3981.8 | 1688.9 ±5347.5 |  | 1381.8 ±4463.9 |  |
| **Visit number of outpatient clinic use,** median (IQR) | | | | | | |
| **All-cause visits** | | 34 (18-61) | 29 (15-52) | <0.001 | 20 (10-35) | <0.001 |
|  | mean±SD | 47.44 ±50.44 | 39.39 ±39.56 |  | 26.11 ±24.68 |  |
| **Mental-health-not-related visits** | | 32 (17-56) | 25 (13-46) | <0.001 | 20 (10-35) | <0.001 |
|  | mean±SD | 43.96 ±47.35 | 34.69 ±35.80 |  | 26.11 ±24.68 |  |
| **Visit number of acute care use,** median (IQR) | | | | | | |
| **All-cause visits** | | | | | | |
| **Emergency department** | | 0 (0-0) | 0 (0-0) | <0.001 | 0 (0-0) | <0.001 |
|  | mean±SD | 0.10 ±0.55 | 0.07 ±0.34 |  | 0.03 ±0.18 |  |
|  | 0 | 39310 (93.1%) | 39728 (94.1%) |  | 41279 (97.7%) |  |
|  | ≥1 | 2932 (6.9%) | 2514 (5.9%) |  | 963 (2.3%) |  |
| **Inpatient** | | 0 (0-1) | 0 (0-1) | <0.001 | 0 (0-1) | <0.001 |
|  | mean±SD | 0.66 ±1.39 | 0.59 ±1.25 |  | 0.36 ±0.79 |  |
|  | 0 | 26951 (63.8%) | 27696 (65.6%) |  | 31628 (74.9%) |  |
|  | ≥1 | 15291 (36.2%) | 14546 (34.4%) |  | 10614 (25.1%) |  |
| **Mental-health-not-related visits** | | | | | | |
| **Emergency department** | | 0 (0-0) | 0 (0-0) | <0.001 | 0 (0-0) | <0.001 |
|  | mean±SD | 0.09 ±0.54 | 0.07 ±0.33 |  | 0.03 ±0.18 |  |
|  | 0 | 39431 (93.4%) | 39874 (94.4%) |  | 41279 (97.7%) |  |
|  | ≥1 | 2811 (6.6%) | 2368 (5.6%) |  | 963 (2.3%) |  |
| **Inpatient** | | 0 (0-1) | 0 (0-1) | <0.001 | 0 (0-1) | <0.001 |
|  | mean±SD | 0.65 ±1.38 | 0.57 ±1.24 |  | 0.36 ±0.79 |  |
|  | 0 | 27237 (64.5%) | 28154 (66.7%) |  | 31628 (74.9%) |  |
|  | ≥1 | 15005 (35.5%) | 14088 (33.3%) |  | 10614 (25.1%) |  |

IQR, interquartile range

**Table S2. Healthcare System Utilization Costs and Visit Numbers in the Three Years Before Initial Diagnosis: Comparison Among Patients with Somatic Symptom and Related Disorders, Depressive Disorders, and Individuals with no Mental Disorder in the age-sex matched samples, aged above 48 (median age)**

|  | | **SSRDs group** | **Depression group** |  | **No mental disorder group** | |
| --- | --- | --- | --- | --- | --- | --- |
| Healthcare system utilization | | (N = 41,632) | (N = 41,632) | P value | (N = 41,632) | P value |
| **Medical cost** ($) **per patient,** median (IQR) | | | | | | |
| **Costs by all uses** | | 2142.6 (1048.7 -4093.0) | 2037.6 (972.8-4034.7) | <0.001 | 1020.1 (471.3-2094.7) | <0.001 |
|  | mean±SD | 3296.1 ±5466.1 | 3406.0 ±5245.9 |  | 2021.3 ±4407.6 |  |
| **Costs by non-psychiatric uses** | | 1988.0 (982.1-3820.1) | 1779.8 (832.6-3593.2) | <0.001 | 1020.1 (471.3-2094.7) | <0.001 |
|  | mean±SD | 3065.5 ±5170.9 | 3003.8 ±4588.2 |  | 2021.3 ±4407.6 |  |
| **Visit number of outpatient clinic use,** median (IQR) | | | | | | |
| **All-cause visits** | | 62 (35-101) | 55 (30-91) | <0.001 | 33 (18-53) | <0.001 |
|  | mean±SD | 78.38 ±68.63 | 69.93 ±63.34 |  | 40.39 ±34.74 |  |
| **Mental-health-not-related visits** | | 59 (33-96) | 50 (27-84) | <0.001 | 33 (18-53) | <0.001 |
|  | mean±SD | 74.24 ±65.52 | 64.49 ±60.40 |  | 40.39 ±34.74 |  |
| **Visit number of acute care use,** median (IQR) | | | | | | |
| **All-cause visits** | | | | | | |
| **Emergency department** | | 0 (0-0) | 0 (0-0) | <0.001 | 0 (0-0) | <0.001 |
|  | mean±SD | 0.09 ±0.39 | 0.08 ±0.34 |  | 0.03 ±0.18 |  |
|  | 0 | 38869 (93.4%) | 39045 (93.8%) |  | 40614 (97.6%) |  |
|  | ≥1 | 2763 (6.6%) | 2587 (6.2%) |  | 1018 (2.4%) |  |
| **Inpatient** | | 0 (0-1) | 0 (0-1) | <0.001 | 0 (0-1) | <0.001 |
|  | mean±SD | 0.87 ±1.57 | 0.92 ±1.76 |  | 0.42 ±1.06 |  |
|  | 0 | 23716 (57.0%) | 23172 (55.7%) |  | 30525 (73.3%) |  |
|  | ≥1 | 17916 (43.0%) | 18460 (44.3%) |  | 11107 (26.7%) |  |
| **Mental-health-not-related visits** | | | | | | |
| **Emergency department** | | 0 (0-0) | 0 (0-0) | <0.001 | 0 (0-0) | <0.001 |
|  | mean±SD | 0.09 ±0.39 | 0.07 ±0.32 |  | 0.03 ±0.18 |  |
|  | 0 | 38924 (93.5%) | 39172 (94.1%) |  | 40614 (97.6%) |  |
|  | ≥1 | 2708 (6.5%) | 2460 (5.9%) |  | 1018 (2.4%) |  |
| **Inpatient** | | 0 (0-1) | 0 (0-1) | 0.011 | 0 (0-1) | <0.001 |
|  | mean±SD | 0.86 ±1.56 | 0.90 ±1.75 |  | 0.42 ±1.06 |  |
|  | 0 | 23902 (57.4%) | 23611 (56.7%) |  | 30525 (73.3%) |  |
|  | ≥1 | 17730 (42.6%) | 18021 (43.3%) |  | 11107 (26.7%) |  |

IQR, interquartile range

**Table S3. Healthcare System Utilization Costs and Visit Numbers in the Three Years Before Initial Diagnosis: Comparison Among Patients with Somatic Symptom and Related Disorders, Depressive Disorders, and Individuals with no Mental Disorder in the age-sex matched samples, men**

|  | | **SSRDs group** | **Depression group** |  | **No mental disorder group** | |
| --- | --- | --- | --- | --- | --- | --- |
| Healthcare system utilization | | (N = 31,603) | (N = 31,603) | P value | (N = 31,603) | P value |
| **Medical cost** ($) **per patient,** median (IQR) | | | | | | |
| **Costs by all uses** | | 1338.6 (563.9-2900.5) | 1161.8 (464.9-2669.1) | <0.001 | 621.6 (248.0-1445.8) | <0.001 |
|  | mean±SD | 2408.9 ±4904.0 | 2548.6 ±7317.0 |  | 1456.4 ±4878.1 |  |
| **Costs by non-psychiatric uses** | | 1203.1 (512.5-2614.1) | 933.6 (364.1-2228.4) | <0.001 | 621.6 (248.0-1445.8) | <0.001 |
|  | mean±SD | 2160.3 ±4459.0 | 2059.2 ±6619.3 |  | 1456.4 ±4878.1 |  |
| **Visit number of outpatient clinic use,** median (IQR) | | | | | | |
| **All-cause visits** | | 36 (18-67) | 29 (13-55) | <0.001 | 19 (9-37) | <0.001 |
|  | mean±SD | 51.82 ±56.83 | 42.45 ±48.96 |  | 26.96 ±27.83 |  |
| **Mental-health-not-related visits** | | 34 (17-62) | 25 (11-49) | <0.001 | 19 (9-37) | <0.001 |
|  | mean±SD | 48.36 ±53.79 | 37.63 ±45.52 |  | 26.96 ±27.83 |  |
| **Visit number of acute care use,** median (IQR) | | | | | | |
| **All-cause visits** | | | | | | |
| **Emergency department** | | 0 (0-0) | 0 (0-0) | <0.001 | 0 (0-0) | <0.001 |
|  | mean±SD | 0.10 ±0.56 | 0.07 ±0.33 |  | 0.03 ±0.18 |  |
|  | 0 | 29501 (93.4%) | 29773 (94.2%) |  | 30883 (97.7%) |  |
|  | ≥1 | 2102 (6.6%) | 1830 (5.8%) |  | 720 (2.3%) |  |
| **Inpatient** | | 0 (0-1) | 0 (0-1) | 0.243 | 0 (0-0) | <0.001 |
|  | mean±SD | 0.66 ±1.42 | 0.69 ±1.58 |  | 0.33 ±0.80 |  |
|  | 0 | 20325 (64.3%) | 20277 (64.2%) |  | 24542 (77.7%) |  |
|  | ≥1 | 11278 (35.7%) | 11326 (35.8%) |  | 7061 (22.3%) |  |
| **Mental-health-not-related visits** | | | | | | |
| **Emergency department** | | 0 (0-0) | 0 (0-0) | <0.001 | 0 (0-0) | <0.001 |
|  | mean±SD | 0.09 ±0.56 | 0.07 ±0.31 |  | 0.03 ±0.18 |  |
|  | 0 | 29564 (93.6%) | 29867 (94.5%) |  | 30883 (97.7%) |  |
|  | ≥1 | 2039 (6.4%) | 1736 (5.5%) |  | 720 (2.3%) |  |
| **Inpatient** | | 0 (0-1) | 0 (0-1) | 0.859 | 0 (0-0) | <0.001 |
|  | mean±SD | 0.65 ±1.41 | 0.67 ±1.56 |  | 0.33 ±0.80 |  |
|  | 0 | 20517 (64.9%) | 20696 (65.5%) |  | 24542 (77.7%) |  |
|  | ≥1 | 11086 (35.1%) | 10907 (34.5%) |  | 7061 (22.3%) |  |

IQR, interquartile range

**Table S4. Healthcare System Utilization Costs and Visit Numbers in the Three Years Before Initial Diagnosis: Comparison Among Patients with Somatic Symptom and Related Disorders, Depressive Disorders, and Individuals with no Mental Disorder in the age-sex matched samples, women**

|  | | **SSRDs group** | **Depression group** |  | **No mental disorder group** | |
| --- | --- | --- | --- | --- | --- | --- |
| Healthcare system utilization | | (N = 42,242) | (N = 42,242) | P value | (N = 42,242) | P value |
| **Medical cost** ($) **per patient,** median (IQR) | | | | | | |
| **Costs by all uses** | | 1847.4 (854.6-3651.1) | 1721.6 (779.8-3477.1) | <0.001 | 915.0 (411.3-2005.5) | <0.001 |
|  | mean±SD | 2913.8 ±4940.5 | 2810.6 ±4055.1 |  | 1846.1 ±4158.9 |  |
| **Costs by non-psychiatric uses** | | 1701.3 (790.1-3353.5) | 1481.8 (654.6-3101.9) | <0.001 | 915.0 (411.3-2005.5) | <0.001 |
|  | mean±SD | 2693.1 ±4743.5 | 2512.4 ±3741.9 |  | 1846.1 ±4158.9 |  |
| **Visit number of outpatient clinic use,** median (IQR) | | | | | | |
| **All-cause visits** | | 53 (29-90) | 47 (26-81) | <0.001 | 30 (16-49) | <0.001 |
|  | mean±SD | 69.43 ±64.20 | 61.86 ±56.94 |  | 36.97 ±32.09 |  |
| **Mental-health-not-related visits** | | 50 (27-84) | 43 (23-74) | <0.001 | 30 (16-49) | <0.001 |
|  | mean±SD | 65.41 ±61.15 | 56.65 ±53.93 |  | 36.97 ±32.09 |  |
| **Visit number of acute care use,** median (IQR) | | | | | | |
| **All-cause visits** | | | | | | |
| **Emergency department** | | 0 (0-0) | 0 (0-0) | <0.001 | 0 (0-0) | <0.001 |
|  | mean±SD | 0.09 ±0.42 | 0.08 ±0.34 |  | 0.03 ±0.19 |  |
|  | 0 | 48678 (93.1%) | 49000 (93.7%) |  | 51010 (97.6%) |  |
|  | ≥1 | 3593 (6.9%) | 3271 (6.3%) |  | 1261 (2.4%) |  |
| **Inpatient** | | 0 (0-1) | 0 (0-1) | 0.001 | 0 (0-1) | <0.001 |
|  | mean±SD | 0.82 ±1.52 | 0.79 ±1.51 |  | 0.42 ±1.00 |  |
|  | 0 | 30342 (58.1%) | 30591 (58.5%) |  | 37611 (72.0%) |  |
|  | ≥1 | 21929 (41.9%) | 21680 (41.5%) |  | 14660 (28.0%) |  |
| **Mental-health-not-related visits** | | | | | | |
| **Emergency department** | | 0 (0-0) | 0 (0-0) | <0.001 | 0 (0-0) | <0.001 |
|  | mean±SD | 0.09 ±0.41 | 0.07 ±0.33 |  | 0.03 ±0.19 |  |
|  | 0 | 48791 (93.3%) | 49179 (94.1%) |  | 51010 (97.6%) |  |
|  | ≥1 | 3480 (6.7%) | 3092 (5.9%) |  | 1261 (2.4%) |  |
| **Inpatient** | | 0 (0-1) | 0 (0-1) | <0.001 | 0 (0-1) | <0.001 |
|  | mean±SD | 0.81 ±1.51 | 0.78 ±1.50 |  | 0.42 ±1.00 |  |
|  | 0 | 30622 (58.6%) | 31069 (59.4%) |  | 37611 (72.0%) |  |
|  | ≥1 | 21649 (41.4%) | 21202 (40.6%) |  | 14660 (28.0%) |  |

IQR, interquartile range
